# Pregnancy Outcomes Complicated by Preterm Premature Rupture of Membranes: Retrospective Review of Cases in Three Institutions in Kazakhstan

**DOI:** 10.5195/cajgh.2014.222

**Published:** 2014-06-15

**Authors:** Balkenzhe Imankulova, Gulzhanat Aimagambetova, Layzzat Saiddildina, Talshyn Ukybassova

**Affiliations:** National Research Center for Maternal and Child Health, Astana, Kazakhstan

**Keywords:** perinatal mortality, perinatal morbidity, pre-term membrane rupture, pre-term delivery

## Abstract

**Introduction:**

Pre-term premature rupture of membranes (PPROM) is one of the leading causes of perinatal morbidity and mortality. This complication is diagnosed in 3% of pregnant women in Kazakhstan, and it is the leading cause of pre-term deliveries. The aim of this study was to determine the outcomes of pregnancies complicated by PPROM in gestation periods between 24 to 32 weeks among three institutions in Kazakhstan.

**Methods:**

This is descriptive analysis of 154 cases with PPROM observed between 24 to 32 weeks of gestation at Perinatal Centers #2 and #3 and the National Research Center for Maternal and Child Health, Astana, Kazakhstan. Cases were selected on the basis of retrospective chart review where PPROM diagnosis occurred in 2013. Descriptive statistics were utilized for data analysis.

**Results:**

The most frequent complications associated with PPROM were threat of miscarriage (13.6% of cases) and chronic placental insufficiency (7.8%). The mean time between PPROM and onset of spontaneous labor was 12.1 ± 2.3 days. Spontaneous labor within 3 days after PPROM started in patients with an amniotic fluid index of 3.0 ± 0.2 cm. Complications experienced by PPROM women during delivery and early postpartum period included: precipitous labor (6.4%), weakness of labor activity (16.2%), atonic hemorrhage (1.2%), and chorioamnionitis (3.2%). 37.6% of newborns in this study were admitted to the Intensive Care Unit. Their health complications included pneumonia (7.7%), conjunctivitis (1.3%), omphalitis and infectious-toxic shock (3.8%), intraventricular hemorrhage (7.8%), and respiratory distress (10.3%).

**Conclusion:**

Thus, preterm rupture of membranes is associated with preterm delivery and an increase of neonatal morbidity. Therefore, it is necessary to find ways to effectively manage PPROM, including developing new techniques to restore the amniotic fluid volume in women experiencing PPROM during 24 to 32 weeks of gestation.

